# Perceptions of Antibiotic Use and Resistance: Are Antibiotics the Dentists’ Anxiolytics?

**DOI:** 10.3390/antibiotics10060735

**Published:** 2021-06-17

**Authors:** Julie Dormoy, Marc-Olivier Vuillemin, Silvia Rossi, Jean-Marc Boivin, Julie Guillet

**Affiliations:** 1 Département de Chirurgie Orale, Faculté d’Odontologie de Lorraine, Université de Lorraine, 54000 Nancy, France; julie.dormoy9@etu.univ-lorraine.fr (J.D.); marc-olivier.vuillemin@hotmail.fr (M.-O.V.); 2 Apemac EA4360, Université de Lorraine, 54000 Nancy, France; silvia.rossi@univ-lorraine.fr; 3 École de Santé Publique, Université de Lorraine, 54000 Nancy, France; 4 Centre d’Investigation Clinique Plurithématique CIC-P Inserm, CHRU de Nancy, 54000 Nancy, France; jm.boivin@chru-nancy.fr; 5 Département de Médecine Générale, Faculté de Médecine, Université de Lorraine, 54000 Nancy, France; 6 AntibioEst, 54000 Nancy, France; 7 Service d’Odontologie, CHRU de Nancy, 54000 Nancy, France

**Keywords:** antibiotics, antibiotic resistance, perception, attitudes, qualitative study, France

## Abstract

Background: Antibiotic resistance is a global health crisis. The aim of this study was to explore dentists’ perceptions of antibiotic resistance. Methods: A qualitative method was used. Seventeen dentists practising in the Nancy (Lorraine, France) region were surveyed. They were general practitioners or specialised in oral surgery, implantology, or periodontology. The practitioners took part in semi-structured interviews between September 2019 and July 2020. All of the interviews were transcribed in full and analysed thematically. Results: Four major themes have been selected: attitudes of the dentists in regard to the guidelines, clinical factors that influence prescriptions, non-clinical factors that influence prescriptions, and the perception of antibiotic resistance. The dentists stated that they were very concerned regarding the public health issue of antibiotic resistance. However, they often prescribe according to their own interests and habits rather than according to the relevant guidelines. Conclusions: Although dentists are generally well aware of antibiotic resistance, they often do not adequately appreciate the link between their prescribing habits and the phenomenon of antibiotic resistance. Regular updating of practitioners’ knowledge in this regard is necessary, but patients and the general public should also be made more aware of the issue.

## 1. Introduction

Antibiotic resistance represents a global public health crisis that has the potential to lead to numerous morbidities and increased mortality in the next 10 years due to the emergence of multi-resistant strains [1,2]. Repeated or prolonged exposure of bacteria to antibiotics allows them to develop defense mechanisms and thereby become resistant to antibiotics. Antibiotic resistance is, therefore, accelerated by repeated administration of antibiotics and their misuse [3,4].

In France, antibiotics are mainly prescribed for outpatients (93%), and general practitioners are responsible for 70% of all antibiotic prescriptions [5]. Dentists generate a significant proportion of these prescriptions, estimated at 12.1% of all antibiotic prescriptions in France [6] and approximately 10% worldwide [7].

Numerous global [8], European [9], and national [10] strategies have been implemented to combat overprescription of antibiotics, targeting both health professionals and the general public. Indeed, a modicum of awareness is necessary among health professionals, as well as among the general population, to reduce antibiotic resistance.

It is not currently known to what extent French dentists are aware of antibiotic resistance. For those who are, it is not known how they perceive their share of responsibility in this phenomenon.

The aim of this study was to explore dentists’ perceptions of antibiotic resistance. To do this, dental practitioners’ use of antibiotics and their position regarding the guidelines for antibiotic prescription were studied.

## 2. Results

Twenty of the 40 dentists who were contacted agreed to participate in the study. The interviews took place between September 2019 and July 2020. The interviews were discontinued after 17 encounters as data saturation was reached. The characteristics of the dentists interviewed are summarised in Table 1. The interviews lasted between 9 and 53 min, with a median duration of 20 min.

### 2.1. Position in Relation to the Guidelines

More than half of the practitioners (13/17) were supportive of the antibiotic prescription guidelines, and they stated that they used them in their practice. Nevertheless, some of the practitioners expressed the opinion that the recommendations sometimes do not adequately take into account the clinical reality (P9). The three practitioners who had a university hospital practice in addition to their private practice stated that this aspect of their practice provided them with a source of information and allowed them to remain informed (P8).

“*It’s a consensus of college professors who don’t practice. I’m sorry, it’s not to say that I am unaware of the issues …*”

P9

“*Honestly, if I wasn’t in the hospital, I wouldn’t have done any…well…I don’t think I would have known about it*”

P8

The majority of the implant practitioners (8/9) reported that they routinely prescribe antibiotics during their surgeries and they claimed to be aware of the guidelines. They justified their prescriptions by a desire to prevent adverse effects from infection or even to reassure themselves. All of the dentists practising implantology prescribed a 7-day course of antibiotics, except for one dentist who recommended antibiotic prophylaxis before the procedure (P13).

“*It’s true that empirically, I haven’t seen any significant difference after surgery with or without antibiotics. But for my own peace of mind, I like to give them a dose of antibiotics beforehand anyway*”

P13

More than half of the practitioners (11/17) felt that there was a degree of discrepancy between the guidelines and the actual prescriptions issued by the dentists. However, not all of the dentists interviewed (4/17) considered themselves as being responsible for this discrepancy. The main reason given for the discrepancy in their professional environment was the lack of information and training regarding the guidelines. This, they said, was either because information about the guidelines was buried among other information or had to be searched for in order to obtain it (P9). Amoxicillin was the antibiotic of choice for most of the practitioners. This drug was sometimes even considered not to be a potential source of antibiotic resistance (P17). However, the combination of spiramycin and metronidazole was a source of disagreement. While some considered this association to be obsolete and ineffective, most of the practitioners still used it in their practice, sometimes even describing it as indispensable to the profession (P9).

“*There should be regular reminders. Because when the recommendations were made, I didn’t hear anything about them. It’s not a subject that interests me when I go to a conference*”

P9

“*I don’t feel like I have a very harmful role in terms of public health, just by prescribing amoxicillin*”

P17

“*With Birodogyl^®^ (i.e., spiramycin + metronidazole) we have fewer good results, but I sometimes give it because it’s quite well-tolerated, and it is a way to provide metronidazole*”

P9

### 2.2. Clinical Factors That Influence Prescriptions

All of the practitioners referred to how antibiotic prescriptions are necessary in their daily practice in order to prevent or treat infections. They all reported taking into account the risks associated with certain treatments, such as bisphosphonates, radiation therapy, or cancer chemotherapy, as well as the risks associated with chronic illnesses. Some practitioners tended to routinely prescribe antibiotics to prevent complications (P9). Others readily referred patients to the hospital when they perceived them to be at high risk, as they did not feel confident enough to manage these cases and their potential complications (P11). Finally, some dentists instead sought the advice of the general practitioner or the specialist in charge of the patient to make a decision.

“*I find it more difficult than before, because in some cardiology protocols you now have to give antibiotics, in others you don’t have to. So, I tend to give them systematically when people are at risk ok bacterial diffusion*”

P9

“*Uh… (lowered voice) I address. […] yes, because it’s not so much that we don’t know how what is involved, it’s that we ultimately have to manage the complications, and in private practice, we don’t have the means to manage it, in any case, they don’t give them to us*”

P11

### 2.3. Non-Clinical Factors That Influence Prescriptions

The majority of the practitioners admitted that they sometimes prescribe antibiotics for their own peace of mind. Indeed, they tended to prescribe an antibiotic in case of doubt (P8), to prevent possible complications following treatments (infections, bone, pain, etc.), or out of fear of negative feedback or even medico-legal action (P17). At times, they were able to prescribe antibiotics in cases where they were not indicated but the patients requested them. Lack of time, often in the context of emergency situations, tended to result in relatively frequent prescription of antibiotics. In addition, the prescription of antibiotics was sometimes thought to be warranted for patients who were managed just before the weekend or before going on vacation (P14). More personal factors could influence what the dentists prescribed, such as their family and friends or their habits. Their clinical experience, or even significant one-off experiences, tended to result in new prescribing habits. Some of the younger dentists considered that older practitioners tended to overprescribe antibiotics (P4).

Most of the participants felt that the patients had a pronounced influence on what they prescribed. A few dentists stated that they prescribe antibiotics for the sake of patient comfort, their “psychological need”, or to reassure their patients, particularly in case of patients who they considered to be very demanding and insistent (P1). Others claimed that the patient’s age was a factor and they tended to prescribe more antibiotics for older or very young patients. One practitioner felt that young patients did not always adhere to prescription durations and they, therefore, tended to prescribe antibiotics for relatively short durations.

“*For some people, for peace of mind, you put down 6 days of amoxicillin. That’s not going to create resistance and then at least I’m off the hook*”

P8

“*In France, we have this notion that in all cases we must take precautions, and that if you have prescribed antibiotics, then you can’t be blamed for not having given any*”

P17

“*On Fridays, I’m going to do it all day long because, if there is a chance of a problem, I want to prevent it from happening. I don’t want the guy to have to go back to the dentist who is on call to get a prescription, so it depends a bit on the day of the week*”

P14

“*When all is said and done, I think we are still part of the generation where we already know that antibiotics are no longer a silver bullet. Unlike the generation that preceded us, for whom antibiotics were a novelty and a marvelous invention that could seemingly cure anything*”

P4

“*I’m going to reserve it for the patients who are difficult (laughs), patients who don’t let up; then -in case of any doubt- I usually throw in some antibiotics, anti-inflammatories, and analgesic so that they stop bothering me*”

P1

### 2.4. Perception of Antibiotic Resistance by Dentists

The main idea highlighted was a sense of awareness regarding antibiotic resistance, despite a lack of clinical hindsight and direct confrontation of participants in their practice (P15). A majority of practitioners acknowledged that dentists were also implicated to a certain extent in the phenomenon of antibiotic resistance, but they nonetheless did not believe that they played a major role in this phenomenon (P2). Despite this general concern, the participants did not feel that they were sufficiently informed regarding the subject, as they felt that the subject was not sufficiently discussed either in training courses or in professional journals.

A few dentists felt that patients have a large degree of responsibility, particularly in terms of non-compliance or sometimes even self-medication (P3). According to some of the practitioners, this was due to the lack of understanding among patients, i.e., a lack of knowledge regarding antibiotics and the phenomenon of antibiotic resistance. A collective increased awareness of patients was thought to be necessary (P10). One practitioner said that dentists even have a role in informing patients about the misuse of antibiotics.

While the dentists acknowledged that they have a degree of responsibility in regard to the development of antibiotic resistance, many of the participants identified physicians as being the main culprits, particularly general practitioners. Indeed, they were often reproached for prescribing antibiotics in cases where it was not indicated or involved unsuitable drugs (P13). According to a number of the dentists (P6), hospital medicine, particularly the emergency services, also shared some of the responsibility Some of the participants suggested that pharmacists were also involved in this phenomenon, as the pharmacists’ advice to patients sometimes was not in keeping with their prescriptions (P8).

“*So, it’s true that I feel a little far removed from that, even though I know that it’s a notion that exists, that is proven, that it’s been demonstrated that there is resistance, but it’s true that I feel…yeah, really quite far removed from that*”

P15

“*Oh well, no, I think that we, as dentists, have a fabulous impact on reducing resistance in general, I don’t think we are the ones to blame*”

P2

“*Some people self-medicate or stop treatment, and this leads to antibiotic resistance*”

P3

“*I don’t think patients are aware of this. I think details regarding the medication are a little bit overwhelming for them. Most of them don’t even know why they’re taking antibiotics. So, from there to understanding antibiotic resistance…*”

P10

“*But it’s true that the typical doctor’s prescription when it comes to teeth is to prescribe NSAIDs (i.e., non-steroidal anti-inflammatory drugs) and Birodogyl^®^ (i.e., spiramycin + metronidazole), systematically.*”

P13

“*In my opinion, it’s more in general medicine and even in the hospital. How many people go to the emergency room and get antibiotics because they have a toothache?*”

P6

*(speaking of pharmacists) “They put ideas into the heads of patients who are already lacking confidence*”

P8

## 3. Discussion

Dentists have a significant role to play in the fight against antibiotic resistance, not only because of the number of prescriptions that they issue but also because of their relevance. This work, which to our knowledge is the first of its kind in France, highlights a number of previously undescribed non-clinical factors that influence the prescription of antibiotics. It also reveals how French dentists perceive antibiotic resistance.

An antibiotic prescription needs to be justified by the existence of an infection, identifiable by specific clinical signs, or for prevention during certain high-risk procedures, particularly in patients who are in poor health [11]. However, rather than relying on objective clinical criteria, dentists sometimes wish to reassure themselves, as well as sometimes their patients, with excessive prescriptions based on non-clinical factors. In such cases, antibiotic prescription is nonetheless still perceived as being essential. A parallel can be drawn with general medicine: for example, the use of antibiotics by doctors in case of a fever [12] seems comparable to the use of antibiotics by dentists in case of exacerbated pain [13].

A discrepancy between the statements regarding the knowledge of the current French guidelines and their applications has already been highlighted in a recent article [14]. The practitioners interviewed for the qualitative study appear to justify this discrepancy, on the one hand, by the difficulty with accessing information and the lack of training on the subject, but also by the fact that to them the guidelines do not appear to adequately take into account the realities of daily clinical practice. This is particularly the case with implant surgery: the majority of the practitioners performing implantology on a recurrent or exclusive basis (dental general practitioners or specialists) use antibiotics mainly for prevention in their practice, with the knowledge that this is neither necessary nor recommended in healthy patients [11,15,16]. In a sense, antibiotics then become “anxiolytics” for these practitioners. Implant surgery represents a significant cost [17] for patients who do not have health insurance coverage, unlike many other procedures in France. The fear of peri-implant infection, and, therefore, treatment failure, could explain these unwarranted prescriptions of antibiotics. This could also be due to the fact that the guidelines have evolved in implantology. Indeed, the prescription of antibiotics was still recommended until 2001 [18]. The knowledge of practitioners not being updated could hence be a factor that influences this misuse. Furthermore, there is not an international consensus regarding the use of antibiotics in the perioperative phase of dental implant placement in healthy patients [16].

The current situation is reason for concern. Indeed, dentists are not sufficiently aware and sensitised to antibiotic resistance. They acknowledge having a degree of involvement in the development of this phenomenon, but they maintain that other health professionals, particularly general practitioners, are the main party responsible. This was already highlighted in a similar study conducted in the UK in 2014 [2]. Surprisingly, general practitioners consider that hospital doctors and dentists are the main culprits for antibiotic resistance [19].

Antimicrobial resistance is a non-palpable phenomenon that dentists as well as other health professionals, who think that they do not personally encounter it in their daily practice, find hard to fathom. This is probably the root cause of the difficulties with the implementation of effective means of combatting antibiotic resistance in dental surgery as well as in other health professions. Dental surgeons would also benefit from improvement of their initial and postgraduate training. They also need easy access to information and new recommendations when they are published. However, informing patients regarding antimicrobial resistance and the scarcity of real needs for antibiotic prescriptions in dentistry is also essential in order to raise individual and collective awareness [2,20].

Amoxicillin is the antibiotic most prescribed by dentists in France, with 20% of their prescriptions being for this antibiotic [6]. We have shown that some practitioners believe that the use of amoxicillin does not lead to antibiotic resistance. Although amoxicillin is one of the entities that generates the least antibiotic resistance, it is still a potential source of it [1,21,22]. Indeed, in 2014, 22% of all antibiotic resistance was attributable to penicillins in Europe [5]. Some practitioners continue to prescribe a combination of metronidazole and spiramycin, despite its absence from the first-line recommendations [11]. Furthermore, it has been shown that the combination marketed in France was not dosed sufficiently to have an acceptable level of efficacy, since it combines 1.5 M IU of spiramycin and 250 mg of metronidazole. The manufacturer recommends a dosage of two to three tablets per day, leading to a maximum dose of 4.5 M IU of spiramycin and 750 mg of metronidazole. However, the recommended dosage for the treatment of adult infections is 6 to 9 M IU/24 h for spiramycin and 1 to 1.5 g of metronidazole per day. Dentists are the main prescribers of this combination of antibiotics in France, accounting for 63% of all prescriptions [6].

This work has many strengths. Although there have been many qualitative studies at the national [14,23] and international level [24,25] regarding antibiotic prescriptions in dentistry, this study is the first qualitative research in France on this subject. This approach has enabled us to explore the perception of antibiotic resistance by dentists. The results obtained can be compared with those obtained in similar studies conducted worldwide [2,26]. We note similarities in the perception of antibiotic resistance by practitioners in different countries, particularly the predominant responsibility attributed to general practitioners in regard to this phenomenon [2]. Nevertheless, while some factors that influence prescriptions are similar (patient pressure, fear of medico-legal procedures), others differ between countries. For example, in Australia, the socio-economic status of the patient, pressure from dental practice staff, or fear of negative opinions online have been described [26]. In the UK, the unwillingness of patients to receive the appropriate technical procedure is a key factor in antibiotic prescriptions [27]. In France, the day of the week of the consultation and the age and the medical history of patients are factors that influence the prescription of antibiotics. In addition, this study takes a new look at the perception of antibiotic use and antibiotic resistance in implantology practice. One of the many criteria for the success of this study was the participants’ lack of judgment and the semi-structured nature of the interviews, which allowed the dentists to freely discuss the issue and thereby allow new elements to emerge. This would not have been possible with quantitative research. By ensuring their anonymity, the practitioners appeared to be very honest and spontaneous in their responses.

However, this study has certain limitations. Indeed, a desirability toward bias may exist in the responses of the participants, who could sometimes have provided answers according to what seemed acceptable to them rather than according to their own habits, as well as in their interpretation of the questions. A selection bias is inevitably present because the participation was voluntary. Indeed, there were no rural dentists who volunteered to participate in this study. However, we have no reason to believe that their answers would have been fundamentally different from the others. As many clinical and non-clinical factors that influence antibiotic prescriptions by dentists have been mentioned, it would seem relevant to further study these factors in a thorough and exhaustive manner.

This study reflects the perceptions of a diverse sampling of dentists that involved different genders, ages, practices, and modes of practice, but it—like all qualitative research—is not intended to be a representative panel of French dentists.

## 4. Materials and Methods

A qualitative study, based on semi-structured interviews, was chosen to achieve the main objective. 

Initially, the interview script was structured into two main themes: antibiotics and antibiotic resistance. The construction of this script and the formulation of the questions were based on similar studies conducted in general medicine [19,28] and dentistry [26].

The target population of the study was dentists who were in private practice at the time of recruitment (excluding private replacements), as this is the main mode of practice in France [29]. The constitution of the sample was carried out by taking care to involve practitioners who belonged to different age groups and with different durations of being in practice, who practised alone or in shared practices (with collaborators and/or associates), who worked with or without a dental assistant, and who were general practitioners and/or who had a specialised practice (periodontology, implantology, oral surgery, or endodontics). All the participants practised in Nancy (Lorraine, France) or in its suburbs, including rural areas. The participants were contacted by telephone prior to the interview process in order to explain the purpose of the study and the methods for carrying out the interviews.

The interviews were conducted face-to-face while the participants were on their own at their respective workplaces. Two researchers (J.D. and M.-O.V.) conducted the interviews. The first researcher interviewed the practitioner and the second researcher acted as an observer, refocusing the interview if necessary. The interviews were conducted with the consent of each participant. The practitioners did not receive any financial compensation for participation. All of the interviews were recorded and transcribed verbatim. Laughter, hesitation, and the facial expressions of each participant were noted. After the transcriptions, a floating reading of the interviews was carried out separately by the two researchers in order to extract verbatim comments. The verbatims were then pooled in order to build a first analysis grid, which was rectified and then further developed after a second reading. Four major themes were selected: position in relation to the guidelines, clinical factors that influence prescriptions, non-clinical factors that influence prescriptions, and the perception of antibiotic resistance by the dentists. A third reading led to final modifications of the grid and then to a horizontal followed by a vertical analysis with a triangulation of the data [30].

The qualitative research methodology complied with the international Consolidated Criteria for Reporting Qualitative Research (COREQ).

## 5. Conclusions

Dentists are aware of the phenomenon of antibiotic resistance, but they do not appear to appreciate the link between their prescribing habits and the occurrence of bacterial resistance to antibiotics. The factors that influence their prescriptions are numerous, and initial and continuing education alone cannot change their practices.

Nevertheless, there is a real need for regular updating of knowledge. While practitioners are generally amenable to training regarding the guidelines of good practice, these guidelines also require broad and clear channels of dissemination. However, changing behaviours is a complex science, as demonstrated in the work of Michie et al. Indeed, many factors, that are often difficult to take into account, can influence practitioners’ decision making and trigger prescribing. The evaluation of a framework after its implementation is therefore crucial in order to judge the effectiveness of the proposed method, if we hope to change practitioners’ behaviour regarding antibiotic prescriptions [31].

The complexity of the phenomenon of antibiotic resistance and the diversity of the parties involved in its occurrence (antibiotic prescribers, patients, public entities, etc.) highlight the need for further studies. The need for training and information expressed by dentists is associated with a requirement for the involvement of other parties, particularly patients, in raising awareness of antibiotic resistance. Various approaches could be explored, such as the implementation of new campaigns for the general public or the development of explanatory leaflets provided by prescribing practitioners or pharmacists.

## Figures and Tables

**Table 1:** Demographic details.

Practitioner	Gender	Age (Years)	Years of Practising	Work Location	Specialisation	Office	Dental Assistant	Comments
P 1	M	59	31 to 40	Urban	GDP	Group	With	Internship supervisor
P 2	M	49	21 to 30	Urban	GDP with Periodontology and Implantology	Group	With	Internship supervisor
P 3	F	44	21 to 30	Urban	GDP	Individual	Without	
P 4	F	37	11 to 20	Urban	GDP	Group	With	
P 5	F	50	21 to 30	Urban	GDP	Group	With	
P 6	M	28	0 to 10	Urban	GDP	Individual	With	
P 7	F	32	0 to 10	Urban	GDP	Group	With	
P 8	F	58	31 to 40	Urban	GDP with Periodontology and Implantology	Group	With	
P 9	M	72	41 to 50	Urban	Periodontology	Group	With	
P 10	F	29	0 to 10	Urban	Oral surgery	Group	With	
P 11	M	39	11 to 20	Urban	Oral surgery	Group	With	
P 12	M	46	11 to 20	Urban	GDP with Surgery and Implantology	Group	With	
P 13	M	37	0 to 10	Urban	GDP with Periodontology and Implantology	Individual	With	
P 14	M	42	11 to 20	Urban	GDP and Endodontics	Group	With	
P 15	M	38	10	Urban	GDP with Periodontology and Implantology	Individual	With	
P 16	M	44	11 to 20	Urban	Periodontology and Implantology	Group	With	
P 17	M	56	31 to 40	Urban	Periodontology and Implantology	Group	With	

GDP = General Dental Practitioner.
